# Upregulated MELK Leads to Doxorubicin Chemoresistance and M2 Macrophage Polarization via the miR-34a/JAK2/STAT3 Pathway in Uterine Leiomyosarcoma

**DOI:** 10.3389/fonc.2020.00453

**Published:** 2020-04-22

**Authors:** Zhiwei Zhang, Chenggong Sun, Chengcheng Li, Xinlin Jiao, Brannan B. Griffin, Samina Dongol, Huan Wu, Chenyi Zhang, Wenyu Cao, Ruifen Dong, Xingsheng Yang, Qing Zhang, Beihua Kong

**Affiliations:** ^1^Department of Obstetrics and Gynecology, Qilu Hospital, Shandong University, Ji'nan, China; ^2^Gynecology Oncology Key Laboratory, Qilu Hospital, Shandong University, Ji'nan, China; ^3^Department of Pathology, Feinberg School of Medicine, Northwestern University, Chicago, IL, United States

**Keywords:** MELK, uterine leiomyosarcoma, chemoresistance, apoptosis, M2 macrophage, JAK2, STAT3

## Abstract

Uterine leiomyosarcoma (ULMS) is the most lethal gynecologic malignancy with few therapeutic options. Chemoresistance prevails as a major hurdle in treating this malignancy, yet the mechanism of chemoresistance remains largely unclear. In this study, we certified MELK as a poor prognostic marker through bioinformatic analysis of the GEO database. Cellular experiments *in vitro* revealed that MELK played an essential role in ULMS cells' chemoresistance and that a high expression of MELK could lead to doxorubicin resistance. mRNA profiling uncovered the pathways that MELK was involved in which led to doxorubicin resistance. MELK was found to affect ULMS cells' chemoresistance through an anti-apoptotic mechanism via the JAK2/STAT3 pathway. miRNA profiling also revealed that upregulated MELK could induce the decrease of miRNA-34a (regulated by JAK2/STAT3 pathway). We detected that MELK overexpression could induce M2 macrophage polarization via the miR-34a/JAK2/STAT3 pathway, contributing to doxorubicin chemoresistance in the tumor microenvironment. OTSSP167, a MELK inhibitor, may increase ULMS sensitivity to doxorubicin. Our investigation could propose novel targets for early diagnosis and precision therapy in ULMS patients.

## Introduction

Uterine leiomyosarcoma (ULMS) is a scarce tumor subtype, with its incidence being nearly 1% of all uterine malignancies. It comprises ~70% of all uterine sarcomas and contributes to a large proportion of uterine cancer deaths (1). The 5-year survival rate of ULMS patients ranges 15–25% overall and 40–70% for stage I and stage II diseases (2). Primary surgical resection is the mainstay of therapy and confers a prognosis advantage (3). ULMS is also commonly insensitive to chemotherapy. In the National Comprehensive Cancer Network, 2019 treatment guidelines for uterine neoplasms, doxorubicin, and gemcitabine/docetaxel remain the most effective regimens in treating a recurrent or advanced disease. However, presently, cytotoxic regimens remain deficient and the 5-year disease-specific survival is <30%. In a phase III trial including women who had ULMS, carcinosarcoma, or other kinds of sarcoma, administering doxorubicin at the concentration of 60 mg/m^2^ showed an objective response among 16% of all enrolled women and in 25% in those who had ULMS. In the same study, the addition of dimethyl triazenoimidazole carboxamide did not significantly improve the treatment response rates (4). Moreover, the objective response rate of gemcitabine/docetaxel did not even reach 25% in other investigations (5–11). The response rates of the present regimens are dismal, with the partial response rate changing from 0 to 33% and the complete response rate varying from 0 to 8% (12). Other therapeutic options have not seemed to yield promising results. Two phase II clinical trials revealed that ULMS patients had no objective response to nivolumab and pembrolizumab, two kinds of selective PD-1 receptor blockers (13, 14). Most retrospective studies of adjuvant radiotherapy (RT) also suggested no appreciable or consistent improvement in the overall survival of ULMS patients (15).

MELK, a well-known oncogenic gene, was first depicted by exploring the gene landscape of progenitor cells in pediatric brain tumors (16). The upregulation of MELK has been detected in many kinds of tumors consisting of gastric cancer, breast cancer, glioblastomas, colorectal cancer, prostate cancer, and melanoma (17–19). In addition, high MELK expression level is related to poor prognosis in breast cancer patients (18). Previous researches have investigated that MELK may affect cell cycle regulation, cell proliferation, apoptosis, and metastasis (20–23).

Tumor-associated macrophages (TAMs), a crucial element of tumor microenvironments (TMEs), play an important role in tumor promotion including tumor growth, immune suppression, angiogenesis, chemoresistance, and metastasis. Tumor cells or microenvironmental cells can secrete factors which can polarize TAMs into M1 or M2 macrophages (24). M1-type macrophages have been characterized by inducible nitric oxide synthase (iNOS), TNF-α, and IL-12, which potentially inhibit tumor progression. M2-type macrophages are featured by arginase 1 (Arg1), CD206, IL-10, and TGF-β, which may facilitate tumor progression (25–27). Prior works illustrate that TAMs have a significant impact on tumor chemotherapy and radiotherapy (28, 29). Moreover, recent studies have shown that M2 macrophages could affect cancer stem cells in both drug resistance and self-renewal through a complex network of growth factors, chemokines, cytokines, and extracellular matrix molecules (29).

Through a bioinformatics analysis of the Gene Expression Omnibus (GEO) database, we first confirmed MELK to be a poor prognostic marker and then aimed to determine its role in ULMS. Our current study specifically investigates the relationship between MELK expression, ULMS TMEs, and doxorubicin resistance. Our findings could propose novel targets for early diagnosis and precision therapy in ULMS patients.

## Materials and Methods

### Patients and Tissue Samples

With institutional review board approval, a total of 27 tumor specimens were collected at Qilu Hospital from patients who had been diagnosed with ULMS according to the World Health Organization criteria. A total of 27 ULMS, 24 myometrium (MM), and 40 uterine leiomyoma (ULM) cases were included in the study. The tissues were evaluated by two pathologists blinded to clinical data. The International Federation of Gynecology and Obstetrics (FIGO) 2014 staging system was used to categorize the histological grade and the tumor stage. The clinical information and pathological data are shown in Supplementary Table 1.

### Tissue Microarrays and Immunohistochemistry Staining

The LMS TMAs were established by acquiring a 1-mm-diameter core at a representative area in each tumor. The TMA block was built on a semiautomatic tissue arrayer (MiniCore, UK).

Immunohistochemistry was conducted on histological sections of formalin-fixed, paraffin-embedded LMS tissue samples in TMAs. The sections were deparaffinized and rehydrated by xylene and ethanol. Antigen was retrieved with EDTA buffer (pH = 8.0) at 98°C for 15 min. Then, 3% hydrogen peroxide was used to block the endogenous peroxidase, and nonspecific binding was obstructed with goat serum. The primary antibody anti-MELK (1:500 dilution, HPA017214, Sigma Aldrich) was incubated overnight in a humid chamber at 4°C. Expression was visualized by I-View 3,3amberilution, HPA0 staining detection.

### RNA Extraction and Real-Time Quantitative PCR

TRIzol reagent (Invitrogen, USA) was used to extract total RNA. MicroRNAs (miRNA) was synthesized using the One Step PrimeScript miRNA cDNA Synthesis Kit (Takara, JAPAN). SYBR green Premix Ex Taq II (Takara, Japan) was used to conduct RT-qPCR. *U6* was used as the endogenous standard reference gene. The primer sequence used for miR-34a was TGGCAGTGTCTTAGCTGGTTGT.

### Protein Isolation and Western Blotting

RIPA buffer consisting of PMSF, NaF, and Na_3_VO_4_ was used to lyse cells. A BCA Protein Assay kit (Thermo Scientific, USA) was used to quantify the concentration of protein in the cell lysates. The protein samples were divided on 10% SDS-PAGE gels and transferred on 0.22-μm polyvinylidene fluoride membranes (Merck Millipore, USA). Then, 5% skimmed milk was used to block the membranes for 1–2 h. The membranes were incubated overnight with primary antibodies at 4°C. On the following day, horseradish peroxidase-labeled secondary antibodies were used to incubate the membranes for 1.5 h at room temperature. The reactive proteins were then detected with an enhanced chemiluminescence system (GE, USA). GAPDH was used as the endogenous control.

### Cell Lines and Cell Culture

The SK-UT-1 cells and SK-UT-1B cells were of ULMS cell lines. They were acquired from the American Type Culture Collection (ATCC). The HEK293T cells were purchased from the Chinese Academy of Sciences (Shanghai, China). Eagle's Minimum Essential Medium and Dulbecco's Modified Eagle Medium were used to culture ULMS cells and HEK293T cells, respectively. The THP1 cells received from ATCC were cultured in RPMI-1640 medium. Then, 100 ng/mL phorbol myristate acetate (PMA; Sigma-Aldrich, USA) was used to incubate the THP1 cells for 24 h to induce macrophage differentiation. All the cells were maintained at 37°C with 5% CO_2_ in a humidified incubator.

### Plasmid Construction and Lentivirus Production

MELK shRNA in pLKO-puro was obtained from Sigma-Aldrich. PCR was used to expend the coding sequence of MELK, which was then inserted into a pLenti-C-Myc-DDK-IRES-Puro (PCMV) vector (Origene, USA). Then, the HEK293T cells were used to produce lentivirus by transfecting pMD2.G, psPAX2, and constructive vectors. The lentivirus was used to infect the LMS cells for 24 h, and then the cells were chosen for 1 week in a medium including 2 μg/mL puromycin (Merck Millipore, USA). The alive cells were stable-expression cells.

### Cell Proliferation Assay

3-[4,5-Dimethylthiazol-2-yl]-2,5-diphenyl tetrazolium bromide (MTT) assay was used to measure cell proliferation. The cells were planted in 96-well-plates at densities of 1,000 cells per well in quintuplicate. Then, the cells were incubated with doxorubicin at a concentration of 8 nM. At assigned monitoring times, we added 20 μL 0.5 mg/mL MTT (Sigma-Aldrich, USA) to each well and incubated the cells with MTT for 4 h. Later, the supernatants were carefully discarded and 100 μL of dimethyl sulfoxide (Sangon Biotech, China) was added to each well. The absorbance value at 490 nm was evaluated by a microplate reader (Thermo Scientific, USA).

### Cytotoxic Assay

The MTT method was also used in the cytotoxic assay. The cells were seeded in 96-well-plates at densities of 3,000 cells per well in quintuplicate and exposed to doxorubicin (S1208, Selleckchem, USA) at various final concentrations (0, 16, 32, 64, 128, and 256 nM) for 72 h. The MTT reagent was used to estimate the final cell viability, and then we calculated the surviving fractions.

### Clonogenic Assay

SK-UT-1 and SK-UT-1B cells with MELK knockdown or overexpression were seeded in six-well-plates at densities of 1,000 cells per well of SK-UT-1 and 1,500 cells per well of SK-UT-1B, treated with various concentrations of doxorubicin (0, 30, and 60 nM, respectively), and cultured for 2 weeks. Methanol was used to fix the colonies, and 0.1% crystal violet was used to stain the colonies. We took the colonies with more than 50 cells into account.

### Apoptosis Assay

To evaluate the extent of apoptosis, MELK overexpressing or suppressing ULMS cells were first incubated with doxorubicin at a concentration of 20 nM for 48 h. Then, the cells were collected. Propidium iodide and annexin V-FITC (BD Biosciences, USA) were used to stain the collected cells.

### Luciferase Reporter Assay

IL6R is a latent target gene of miR-34a. For wild-type vectors, the 3′ untranslated regions (3′UTR) of IL6R including the binding site were compounded by GenePharma (Shanghai, China) and cloned into pmirGLO vector (Promega Madison, USA). Overlap extension PCR was used to generate mutant constructs. The HEK293T cells were planted in 96-well-plates at densities of 30,000 cells per well, and then the constructive vectors and miR-34a mimics or negative control were co-transfected into the HEK293T cells. At 36 h later, Dual-Glo Luciferase Assay System (Promega Madison, USA) was used to measure the luciferase activity.

### Enzyme-Linked Immunosorbent Assay

A human IL-6 quantikine enzyme-linked immunosorbent assay (ELISA) kit (R&D Systems, USA) was used to measure the concentrations of IL-6 in the cultured media of macrophage cells according to the manufacturer's instructions.

### mRNA and miRNA Sequencing

Both the SK-UT-1 cells overexpressing MELK and the negative control cells were treated with 20 nM doxorubicin for 4 weeks. Then, mRNA and miRNA profiling was conducted both in cells treated with doxorubicin and in cells without treatment.

Each sample's total RNA was used as input material for the small RNA library. The NEBNext® Multiplex Small RNA (SR) Library Prep Set for Illumina® [New England Biolabs (NEB), USA] was used to generate the sequencing libraries following the manufacturer's recommendations. In order to ascribe sequences to each sample, index codes were then added. Briefly, the NEB 3′ SR Adaptor was directly, and specifically, ligated to the 3′ ends of miRNA, siRNA, and piRNA. After the 3′ ligation reaction, the SR RT Primer was crossbred to any excess 3′ SR Adaptor that remained free after the 3′ ligation reaction, ultimately transforming the single-stranded DNA adaptor into a double-stranded DNA molecule. Next, the 5′ ends adapter was ligated to the 5′ ends of miRNAs, siRNA, and piRNA. M-MuLV Reverse Transcriptase (RNase H-) was used to synthesize the initial cDNA strands. LongAmp Taq 2X Master Mix, SR Primer for Illumina, and index (X) primer were used subsequently to perform the PCR amplification. Then, the PCR products were purified. DNA fragments consisting 140–160 bp (the length of small noncoding RNA plus the 3′ and 5′ adaptors) were recovered and dissolved in 8 μL elution buffer. Finally, DNA High Sensitivity Chips was used to assess library quality on the Agilent Bioanalyzer 2100 system. TruSeq SR Cluster Kit v3-cBot-HS (Illumina) was used to conduct the collection of index-coded samples on a cBot Cluster Generation System following the manufacturer's instructions. After cluster generation, the library preparations were sequenced on an Illumina Hiseq 2500/2000 platform, and 50-bp single-end reads were generated.

### Subcutaneous Implanted Tumor and Drug Resistance Assay *In vivo*

The previously cultured SK-UT-1B cells (at a volume of 1 × 10^7^ cells) were resuspended in 200 μL of phosphate-buffered saline and injected subcutaneously into 4- to 5-week-old female NOD.SCID Il2rγ (NSG) mice (NBRI of Nanjing University) on either side of the axilla. After 2 weeks, the tumor-bearing mice were separated randomly into three groups. For the two treatment groups, the first group was treated with only doxorubicin (3 mg/kg/day) injected intraperitoneally, while the other group was treated with both orally fed OTSSP167 (10 mg/kg/day) and doxorubicin (3 mg/kg/day) injected intraperitoneally. The third control group was untreated with any drug. At 4 weeks after the injection, these mice were sacrificed and inspected for growth of subcutaneous tumors.

### Antibodies

The antibodies used include the following: MELK (SIGMA: HPA017214), P53 (Cell Signaling Technology: 48818), phospho-JAK2 (Y1007 + Y1008) (Abcam: ab32101), phosphor-STAT3 (Y705) (Abcam: b76315), STAT3 (Cell Signaling Technology: 30835), IL6 (Proteintech: 21865), IL6R (Proteintech: 23457), BCL2 (Proteintech: 12789), and GAPDH (Cell Signaling Technology: 2118).

### Statistical Analysis

SPSS Statistics 20 software was utilized to perform data analysis. We assessed statistically significant differences between the experimental and the control groups by the use of Student's *t*-test and chi-square test. *P* < 0.05 was regarded as threshold for statistically significant difference (^*^P < 0.05, ^**^P < 0.01).

## Results

### MELK Is Overexpressed in ULMS and Is a Poor Prognosis Marker of Aggressive ULMS

**Figure 1:**
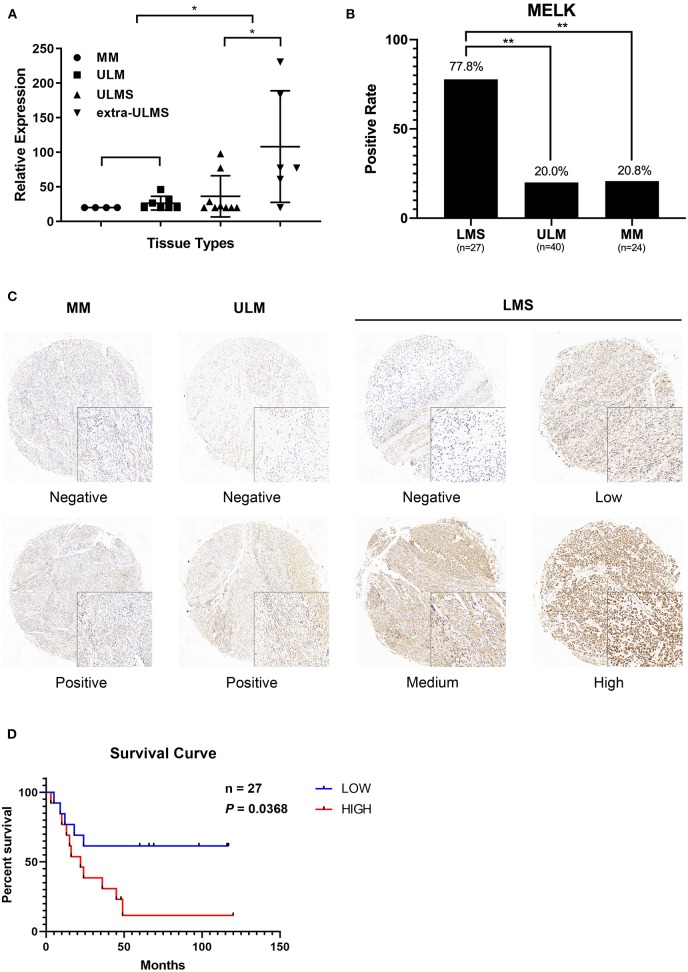
MELK is overexpressed in uterine leiomyosarcoma (ULMS), and its high expression predicts poor prognosis. **(A)** Differential expression of MELK in 15 ULMS samples and seven uterine leiomyoma (ULM) and four myometrium (MM) from GEO data; ^*^P < 0.05. **(B)** The positive rate of MELK expression in ULMS is higher than that in MM and ULM by immunohistochemistry staining; ^**^P < 0.01. **(C)** Representative staining in MM, ULM, and ULMS. **(D)** Overall survival analysis based on MELK expression (high-expression group vs. low-expression group) in our cohort.

According to the data obtained from GEO regarding 15 ULMS samples, seven uterine leiomyoma (ULM), and four myometrium (MM), we detected that MELK expression was significantly overexpressed in ULMS compared to those in ULM and in MM (*P* < 0.01, Figure 1A). Moreover, MELK expression in extreme ULMS was much higher than that in ULMS (*P* < 0.05). We then analyzed MELK protein levels in human ULMS (*n* = 27), ULM (*n* = 40), and MM (*n* = 24). Immunohistochemistry was performed on TMAs. A significantly higher positive rate of MELK expression was seen in ULMS (77.8%, 22/27) than in MM (20.8%, 5/24) and in ULM (20%, 8/40; *P* < 0.01; Figure 1B). Representative staining patterns are displayed in Figure 1C. Furthermore, the survival curves indicated that high MELK expressed in ULMS patients exhibited a significantly shorter overall survival than low MELK expressed in patients (*P* = 0.0368), suggesting that MELK played an important prognostic role in ULMS (Figure 1D). A clinicopathologic feature analysis revealed that MELK expression had no obvious correlation with age, FIGO stage, grade, and tumor size (Table 1). These results suggest that MELK protein is expressed in ULMS and may be expressed at higher levels in some more severe cases.

**Table 1:** Correlation between MELK expression and clinicopathological characteristics in uterine LMS.

**Clinicopathological features**		**Low expression**	**High expression**	***P-*value**
Age (years)	<45	5	7	0.547
	≥45	8	7	
FIGO stage	I + II	8	8	0.816
	III + IV	5	6	
Grade	Low	2	1	0.496
	High	11	13	
Tumor size (cm)	≤5	4	2	0.303
	>5	9	12	

### MELK Can Lead to Doxorubicin Chemoresistance in ULMS Cells

**Figure 2:**
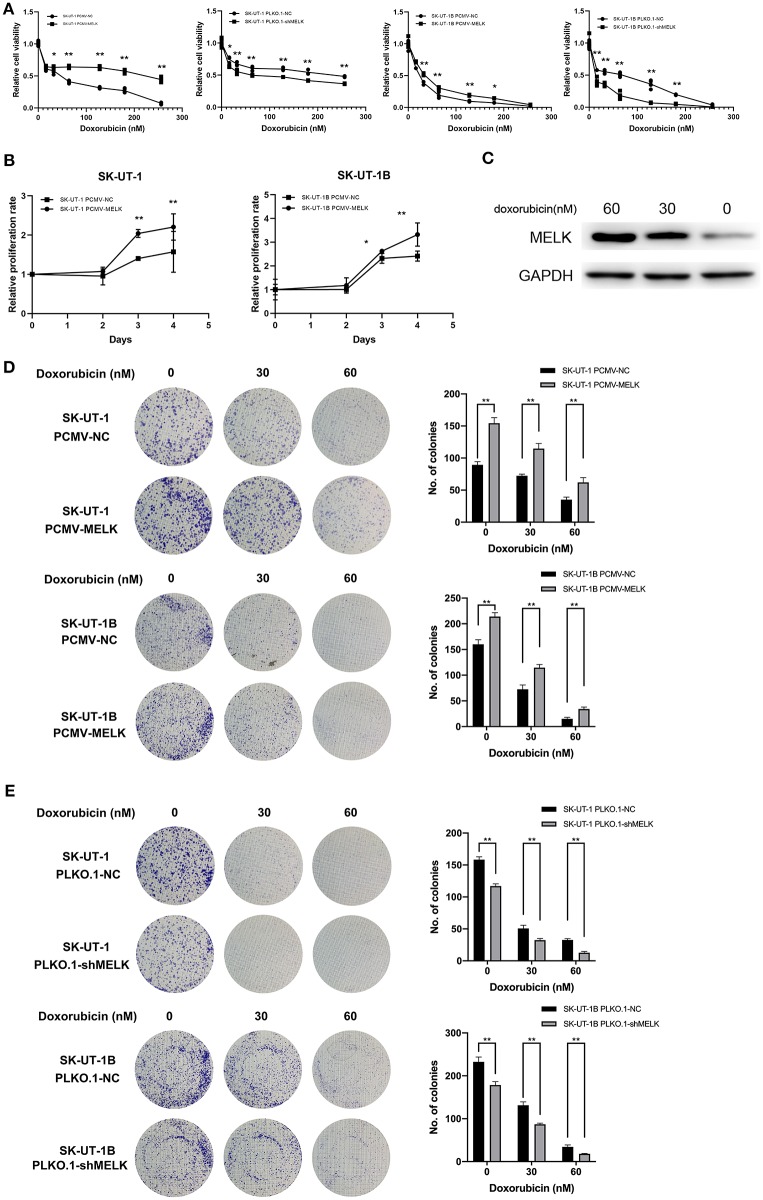
MELK can lead to doxorubicin chemoresistance in uterine leiomyosarcoma (ULMS) cells. **(A)** Cytotoxic assay of SK-UT-1 and SK-UT-1B cells with MELK knockdown or overexpression incubated with different concentrations of doxorubicin (0, 16, 32, 64, 128, or 256 nM) for 72 h; ^*^P < 0.05, ^**^P < 0.01. **(B)** When **c**ells were incubated with doxorubicin at the concentration of 8 nM, the relative viabilities of SK-UT-1 and SK-UT-1B cells overexpressing MELK were significantly higher than that of the negative control group; ^*^P < 0.05, ^**^P < 0.01. **(C)** MELK protein expression was assessed by western blot after 0, 30, or 60 nM doxorubicin was added to the SK-UT-1 cells for 72 h. **(D)** Approximately 1,000 cells/well of SK-UT-1 and 1,500 cells/well of SK-UT-1B were seeded in six-well plates, treated with 0, 30, and 60 nM of doxorubicin, respectively, and cultured for 14 days; ^**^P < 0.01. The results showed that MELK remarkably increased the colony formation in SK-UT-1 and SK-UT-1B cell lines. **(E)** Clonogenic assay of MELK suppressing the ULMS cells showed that MELK suppression apparently decreased the colony formation; ^**^P < 0.01.

Stably transfected ULMS cells overexpressing MELK, those suppressing MELK, and corresponding negative control (NC) cells, respectively, were incubated with doxorubicin at different concentrations (0, 16, 32, 64, 128, and 256 nM) for 72 h. An MTT assay indicated that the ULMS cells' viability could be injured by doxorubicin treatment in a concentration-dependent manner. The relative cell viability of MELK-suppressing cells declined much more rapidly than that of the negative control cells, and the relative viability of MELK-overexpressing cells declined much more slowly than that of the negative control cells at various final concentrations (Figure 2A). Moreover, in the proliferation assay, the relative viability of the ULMS cells with MELK overexpression was significantly higher than that of the negative control cells, especially in the last 2 days (Figure 2B). Analogously, MELK expression remarkably increased colony formation in ULMS cell lines (*P* < 0.05; Figure 2D), and conversely MELK suppression decreased colony formation (*P* < 0.05; Figure 2E). Also, a significantly increased MELK expression in SK-UT-1 cells was seen simultaneously with increasing doxorubicin concentrations (Figure 2C).

### The Effect of MELK on Chemoresistance in ULMS Cells Was Anti-apoptosis via the JAK2/STAT3 Pathway and the Decline of miR-34a

**Figure 3:**
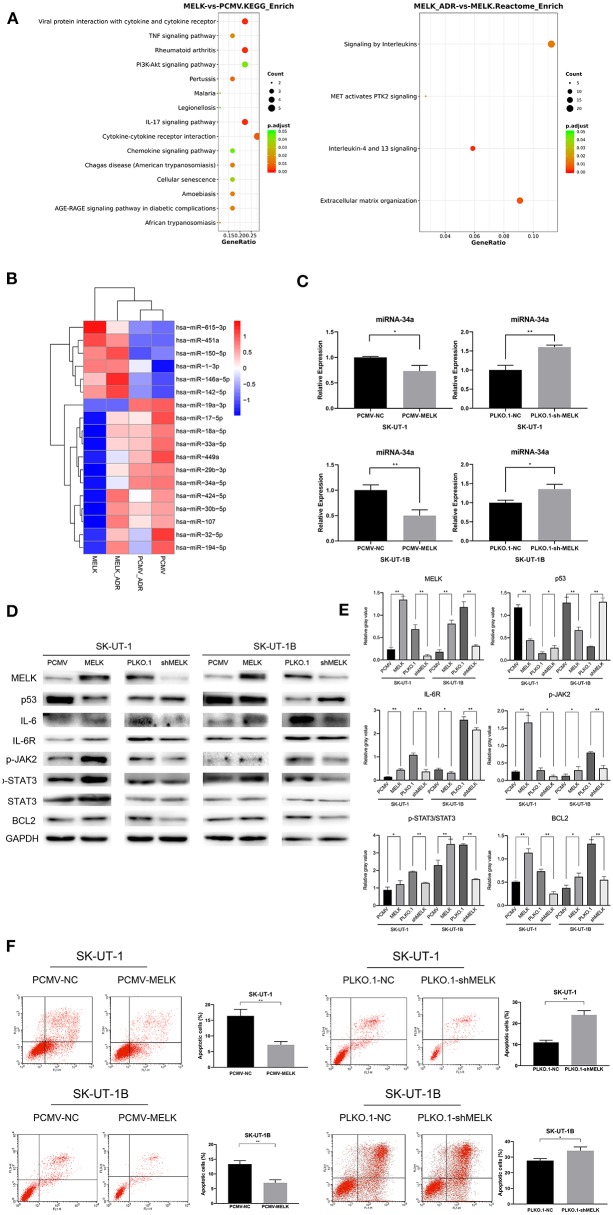
The effect of MELK on uterine leiomyosarcoma (ULMS) cells' chemoresistance was anti-apoptosis via the JAK2/STAT3 pathway and decline of miR-34a. **(A)** Detailed GO enrichment analysis of the pathways in mRNA profiling of SK-UT-1 PCMV-MELK (MELK) vs. SK-UT-1 PCMV-NC (PCMV) and SK-UT-1 PCMV-MELK treated with doxorubicin (MELK-ADR) vs. SK-UT-1 PCMV-MELK untreated (MELK). **(B)** Heatmap of miRNA profiling of SK-UT-1 PCMV-MELK (MELK) vs. SK-UT-1 PCMV-NC (PCMV) and SK-UT-1 PCMV-MELK treated with doxorubicin (MELK-ADR) vs. SK-UT-1 PCMV-NC treated with doxorubicin (PCMV-ADR). **(C)** miRNA-34a expression levels in MELK overexpressing SK-UT-1 and SK-UT-1B cells, MELK suppressed SK-UT-1 and SK-UT-1B cells, and negative control (NC) cells were each assessed by RT-qPCR; ^*^P < 0.05, ^**^P < 0.01. **(D)** JAK/STAT3 pathway markers and BCL2 were evaluated by western blot in SK-UT-1 and SK-UT-1B cells with MELK knockdown or overexpression. [The amino acid positions of phosphorylation were as follows: p-JAK2 (Y1007 + Y1008) and p-STAT3 (Y705)]. **(E)** Statistical analysis of the gray values of the western blot images of each detected protein; ^*^P < 0.05, ^**^P < 0.01. **(F)** Flow cytometric apoptosis assay was evaluated in ULMS cells with MELK knockdown or overexpression treated with doxorubicin at the concentration of 20 nM for 48 h. MELK overexpression inhibited doxorubicin-induced cell apoptosis and MELK knockdown led to more sensitivity to doxorubicin treatment in ULMS cells compared with NC cells; ^*^P < 0.05, ^**^P < 0.01.

To further investigate the mechanism that MELK undertakes in ULMS chemoresistance, SK-UT-1 cells overexpressing MELK and NC cells were treated with 20 nM of doxorubicin for 4 weeks. Next, mRNA and miRNA profiling was conducted in cells both treated with doxorubicin and those without treatment. The results revealed that the upregulated genes in both the MELK-overexpressing cells and the doxorubicin-treated cells were concentrated in pathways such as transcriptional regulation by TP53, signaling by interleukins, interleukins-4 and−13 signaling, and so on. Detailed Gene Ontology (GO) enrichment analysis data of the pathways in mRNA profiling comparing SK-UT-1 PCMV-MELK (MELK) vs. SK-UT-1 PCMV-NC (PCMV) and SK-UT-1 PCMV-MELK treated with doxorubicin (MELK-ADR) vs. untreated SK-UT-1 PCMV-MELK are shown in Figure 3A. Moreover, the miRNA profiling heatmap showed that miR-19a-3p, miR-34a-5p, miR-449a, miR-33a-5p, and miR-29b-3p were suppressed in MELK-overexpressing cells both with and without doxorubicin treatment (Figure 3B). The miRNA profiling results were verified by RT-qPCR. We found that miR-34a level alteration met our expectation. Data revealed not only that MELK could significantly down-regulate miRNA-34a expression in ULMS cells but also that suppressing MELK could apparently upregulate miRNA-34a expression (*P* < 0.05; Figure 3C). It is understood that the JAK2/STAT3 pathway can resist apoptosis and is important in chemoresistance and that STAT3 specifically can decrease the miRNA-34a expression level. Considering this knowledge and our data's findings together, we examined if MELK could promote chemoresistance through the JAK2/STAT3 pathway. Western blot showed that MELK overexpression promoted higher levels of phosphor-JAK2 (Y1007 + Y1008) and phosphor-STAT3 (Y705) (i.e., increased p-JAK2 and the ratio of p-STAT3:STAT3). Conversely, MELK inhibition reduced JAK2 and STAT3 phosphorylation (i.e., decreased p-JAK2 and the ratio of p-STAT3:STAT3). Additionally, the results showed an increase of BCL2 in MELK-overexpressing cells and a decrease of BCL2 in MELK-suppressed cells (Figure 3D). A statistical analysis of the gray values of the western blot images is depicted in Figure 3E. Finally, the apoptosis assay was conducted, and the results indicated that MELK could restrain the apoptosis that is usually induced by doxorubicin treatment (*P* < 0.01; Figure 3F). On account of these results in total, we speculated that MELK regulated the chemoresistance of ULMS cells via the JAK2/STAT3 pathway.

### MELK Can Induce M2 Macrophage Polarization

**Figure 4:**
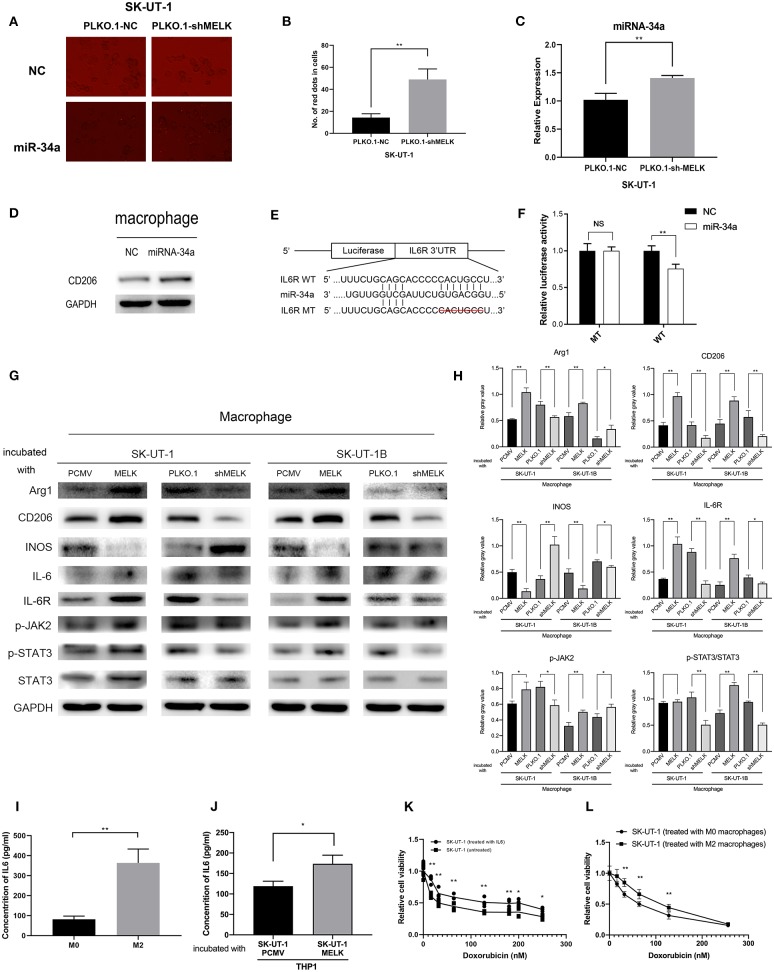
MELK-induced M2 macrophage polarization via the miR-34a/JAK2/STAT3 pathway, and M2 macrophages can secrete IL-6, promoting uterine leiomyosarcoma cells' chemoresistance to doxorubicin. **(A)** SK-UT-1-suppressing MELK and negative control cells were transfected with miRNA-34a mimics with GFP and miRNA-NC. Then, the medium of each cell was used to incubate macrophages. MiR-34a (red dots) was elevated in these macrophages. **(B)** Number of red dots in macrophages that were treated with the conditioned medium of SK-UT-1-suppressing MELK and negative control SK-UT-1 cells; ^**^P < 0.01. **(C)** RT-qPCR of miRNA-34a in exosomes showed that miRNA-34a was significantly higher in the exosome of MELK suppressing SK-UT-1 cells than that of negative control cells; ^**^P < 0.01. **(D)** THP-1 cells were treated with PMA for 24 h and transfected with miR-NC and miR-34a mimics. M2 marker CD206 was significantly increased in miR-34a-transfected macrophages. **(E)** Putative miR-34a binding sequence in the IL6R 3′UTR. The binding sequence of miR-34a in the IL6R 3′UTR sequence was deleted as mutated type. **(F)** Analysis of the luciferase activity of the luciferase reporter plasmid containing either wild-type or mutant IL6R 3′UTR in the HEK 293T cell line which proved that IL6R is the downstream target of miR-34a; ^**^P < 0.01. **(G)** Macrophages were incubated with the conditional media of MELK knockdown, overexpressing or suppressing SK-UT-1 and SK-UT-1B cells and NC cells for 48 h each. Then, macrophage polarization markers and JAK/STAT3 pathway markers were evaluated by western blot in these macrophages. [The amino acid positions of phosphorylation were as follows: p-JAK2 (Y1007 + Y1008) and p-STAT3 (Y705)]. **(H)** Statistical analysis of gray values of western blot images of each detected protein; ^*^P < 0.05, ^**^P < 0.01. **(I)** The macrophages were cultured with IL-4 to promote M2 polarization. Enzyme-linked immunosorbent assay (ELISA) showed that the secreted IL-6 was significantly higher in M2 macrophages (treated with IL-4) than in the normal untreated macrophages; ^**^P < 0.01. **(J)** SK-UT-1 cells were incubated with IL6 (20 ng/mL) and then the treated and untreated cells were measured in cytotoxic assay. The relative cell viability of SK-UT-1 cells treated with IL-6 declined much more slowly than that of the untreated cells; ^*^P < 0.05. **(K)** The macrophages were cultured with a conditioned medium of MELK-overexpressing SK-UT-1 cells and negative control cells for 48 h, respectively. ELISA showed that secreted IL-6 was significantly higher in macrophages treated with a conditioned medium of MELK-overexpressing SK-UT-1 cells than in macrophages treated with a conditioned medium of NC cells; ^*^P < 0.05, ^**^P < 0.01. **(L)** SK-UT-1 cells were cultured with conditioned media of M2 macrophages (treated with IL-4) and normal macrophages. The relative cell viability of SK-UT-1 cells treated with a conditioned medium of M2 macrophages declined much more slowly than that of cells treated with a conditioned medium of normal macrophages; ^**^P < 0.01.

To determine if MELK could induce M2 macrophage polarization, PMA was used to incubate THP-1 cells for 24 h and then the conditioned media of stably transfected ULMS cells with MELK overexpression, ULMS cells with MELK knockdown, and NC cells each *in vitro*. The expression of CD206, Arg1, and INOS (M1 or M2 macrophage markers) was examined by western blot. Compared to NC cells, MELK-overexpressing SK-UT-1 and SK-UT-1B cells showed a significantly increased expression of CD206 and Arg1 as well as a markedly decreased expression of INOS. On the other hand, MELK knockdown SK-UT-1 and SK-UT-1B showed a decreased expression of CD206 and Arg1 as well as an increased expression of INOS (Figure 4G).

### MELK-Induced M2 Macrophage Polarization via miR-34a/JAK2/STAT3 Pathway and M2 Macrophages Can Secrete IL6 Promoting ULMS Chemoresistance to Doxorubicin

Next, we investigated if miR-34a could be transferred to regulate the tumor microenvironment. SK-UT-1 cells with MELK suppression and NC cells were transfected with miRNA-34a mimics with red fluorescent protein and miRNA-NC. Next, the conditioned media from each cell type were used to incubate the macrophages. As displayed in Figures 4A,B, we found that miR-34a was elevated in the macrophages treated with the conditioned medium of SK-UT-1 cells with MELK suppression (*P* < 0.01). We also explored the result of overexpressing miR-34a on macrophage phenotypes. The results of the RT-PCR performed on exosome contents showed that miRNA-34a was significantly higher in the exosomes of SK-UT-1 cells with MELK suppression than in the exosomes of NC cells (*P* < 0.01; Figure 4C). Moreover, PMA was used to incubate THP-1 cells for 24 h, and then the cells were transfected with miRNA-34a mimics and miRNA-NC. Protein level detection showed that the M2 marker CD206 was significantly increased in miR-34a-transfected cells (Figure 4D). A potential miR-34a binding site was identified in the 3′UTR of IL6R (Figure 4E). The 3′UTR segment, including the binding site and its corresponding mutant counterpart, was cloned into pmirGLO vectors, respectively. Then, the vectors were transfected into HEK 293T cells, and the luciferase value differences of the clones were detected ultimately. We confirmed that miR-34a over-expression markedly reduced the luciferase activity in cells transfected with the wild-type 3′UTR binding site but not in cells transfected with the corresponding mutant site, which suggested that miR-34a could bind to IL6R's 3′UTR directly (*P* < 0.01; Figure 4F). Furthermore, we measured the expression of phospho-JAK2 (Y1007 + Y1008) and phospho-STAT3 (Y705) in macrophages treated with the respective conditioned media of stably transfected ULMS cells overexpressing MELK, those suppressing MELK, and corresponding NC cells. Western blots showed a remarkably higher phosphorylation of JAK2 and STAT3 in macrophages cultured with the conditioned medium of ULMS cells overexpressing MELK and a significantly lower phosphorylation of JAK2 and STAT3 in the macrophages cultured with the conditioned medium of ULMS cells suppressing MELK, each compared to the phosphorylation levels in the macrophages conditioned with a medium of NC cells (Figure 4G). A statistical analysis of the gray values of western blot images is depicted in Figure 4H. These results in combination indicated that MELK can induce M2 macrophage polarization via the miR-34a/JAK2/STAT3 pathway.

To investigate how M2 macrophages could promote doxorubicin chemoresistance, macrophages were cultured with IL-4 to promote M2 polarization. The ELISA assessment of IL-6 concentrations revealed that the IL-6 was meaningfully higher in the cultured medium of M2 macrophages treated with IL-4 than in the cultured medium of untreated macrophages (*P* < 0.01; Figure 4I). For the cytotoxic assay, SK-UT-1 cells were treated with IL-6 (20 ng/mL), and the relative viability of both the cells treated with doxorubicin and the untreated cells was measured. The results indicated that the relative cell viability of the SK-UT-1 cells treated with IL-6 declined much more slowly than that of the untreated cells (Figure 4J). To demonstrate that MELK overexpression in ULMS could induce M2 macrophage IL-6 secretion, macrophages were cultured with conditioned media each of MELK-overexpressing SK-UT-1 cells and NC cells for 48 h. Again, ELISA was utilized to measure the IL-6 concentrations. The results showed that IL-6 concentration was expressively higher in macrophages treated with a conditioned medium of SK-UT-1 cells overexpressing MELK than in macrophages treated with a conditioned medium of NC cells (Figure 4K). Additionally, the cytotoxic assay was also conducted on SK-UT-1 cells cultured with a conditioned medium of M2 macrophages (treated with IL-4) and normal macrophages. Relative cell viability was detected, and the results showed that the viability of SK-UT-1 cells treated with conditioned medium of M2 macrophages declined much more slowly than that of the cells treated with the conditioned medium of normal macrophages. Our data suggest that M2 macrophage could contribute to doxorubicin chemoresistance in ULMS cells (Figure 4L).

### OTSSP167 Could Contribute to Doxorubicin's Therapeutic Effect

**Figure 5:**
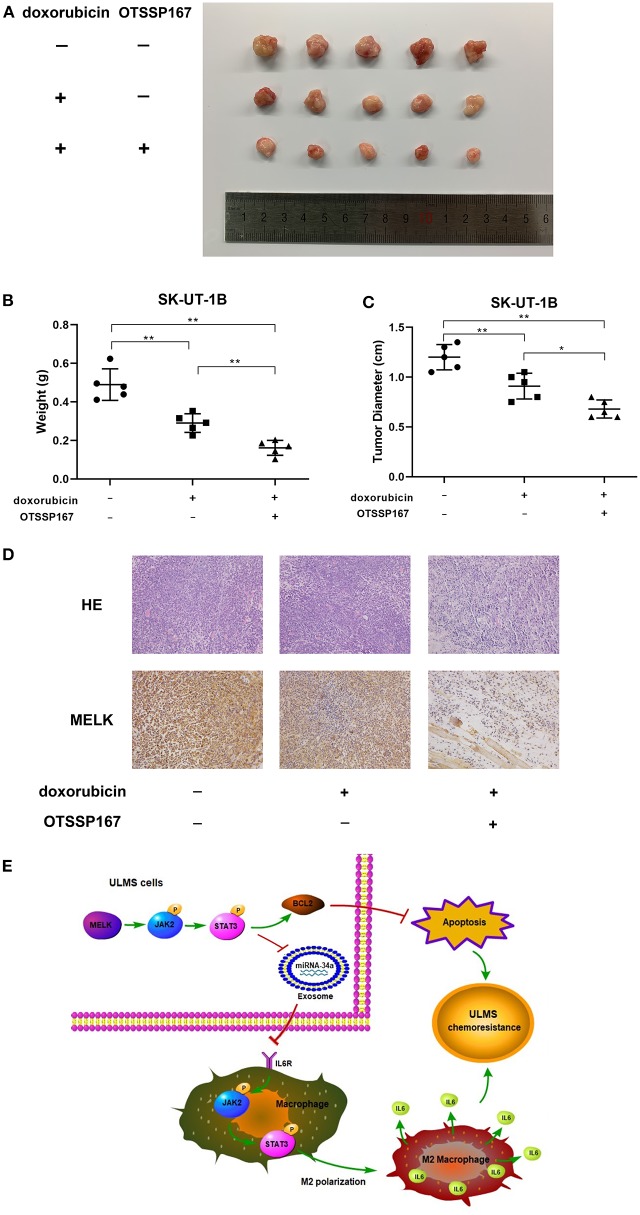
MELK inhibitor OTSSP167 could contribute to the therapeutic effect of doxorubicin. **(A)** The SK-UT-1B cells were injected subcutaneously. Tumor-bearing mice were separated randomly into three groups. One group was treated with doxorubicin only (3 mg/kg/day) intraperitoneally and the other treated group was given both orally fed OTSSP167 (10 mg/kg/day) and doxorubicin (3 mg/kg/day) injected intraperitoneally. The subcutaneous implanted uterine leiomyosarcoma (ULMS) tumors of each group of NCG mice. **(B)** The graphical representation of the tumor weights of each group of NCG mice; ^**^P < 0.01. **(C)** The graphical representation of the tumor size of each group of NCG mice; ^*^P < 0.05, ^**^P < 0.01. **(D)** H&E and MELK immunohistochemical staining were performed in each group of tumors. **(E)** MELK not only affects the chemoresistance of ULMS cells through an anti-apoptotic mechanism via the JAK2/STAT3 pathway but also promotes M2 macrophages' polarization via the same pathway, ultimately contributing to the ULMS cells' chemoresistant ability.

The results of the subcutaneous implanted tumors and drug resistance assay *in vivo* are shown in Figure 5. Our experiment *in vivo* (Figure 5A) affirmed that both doxorubicin alone and the combination of doxorubicin and OTSSP167 could significantly suppress tumor growth, whereas the combination treatment produced the lowest tumor weight (mean tumor weight of the three subgroups: 0.4897 ± 0.03658, 0.2900 ± 0.02153, and 0.1618 ± 0.01732; *P* < 0.01) and tumor size (mean tumor size of the three subgroups: 1.200 ± 0.05701, 0.9100 ± 0.05788, and 0.6800 ± 0.04062; *P* < 0.01; Figures 5B,C). H&E and MELK immunohistochemical staining were performed in each group of tumors and are displayed in Figure 5D.

## Discussion

MELK is a strong oncogenic gene which plays a crucial role in cell proliferation, cell cycle regulation, apoptosis, and metastasis (20–23). Choi and Ku (30) reported that with the treatment of 5-fluorouracil or radiation, the level of MELK expression in rectal cancer cell lines was significantly increased, and their data also suggested that MELK participated in cell cycle, which indicated that MELK potentially acted to mitigate chemotherapy and radiation. Park et al. (31) investigated that FOXM1, an oncogenic transcription factor, and putative substrate of MELK, may sensitize resistant triple negative breast cancer cells to doxorubicin treatment by regulating the DNA damage repair genes. In addition, a study about high-grade gliomas showed that MELK could inhibit radiation-induced apoptosis in glioma stem cells. Additionally, in recurrent high-grade gliomas, cells where MELK is highly expressed accumulated, and they were incapable of response to conventional treatments (23). Moreover, many studies have shown that MELK's phosphorylation of target molecules is an important mechanism of action. Seong and Ha (32) demonstrated that MELK could regulate the stability of p53 by phosphorylating Ser15 in its N-terminal transactivation region, thereby promoting p53-dependent cell cycle arrest and apoptosis. Among the author's studies, another study revealed that MELK binds to and phosphorylates Smad protein and positively regulates TGF-β transcription, thus overall influencing TGF-β mediated apoptosis, growth retardation, and other functions (33).

In the current investigation, the GEO database analysis and immunohistochemistry staining indicated that MELK was a poor prognosis marker of aggressive ULMS and even promoted cellular resistance to doxorubicin. Moreover, the overexpression of MELK induces an increased expression of IL-6 and BCL2. MELK also increases the phosphorylation of JAK2 and STAT3 in ULMS. In previous studies, IL-6 or IL-6R downstream signaling conferred chemoresistance by activating the JAK1/STAT3, MAPK/ERK, or PI3K/AKT pathways (34–36). Supported by knowledge from the literature, our findings indicate that MELK may promote resistance to doxorubicin by triggering the IL-6/JAK2/STAT3 signaling pathway. In solid tumors, including prostate cancer and breast cancer, STAT3 activation which was mediated by IL-6 could enhance the expression of anti-apoptotic proteins, such as BCL2 or survivin which has constantly been regarded as a protective mechanism from chemotherapy-induced cell death (37–39). In our work, BCL2 was increased in cells with MELK overexpression, which strongly suggested that MELK upregulated BCL2 via the JAK2/STAT3 pathway to restrain the apoptosis caused by chemotherapy's cytotoxic effects.

miRNAs are developmental extremely conservative noncoding RNA molecules. Owing to its context-dependent and specific mechanisms, miRNA plays essential roles in large-scale physiological processes, especially in both anti-neoplasia and pro-neoplasia effects in cancer (40). Since tumor cells connect with their microenvironment partially by miRNAs, miRNAs are identified as a method of antineoplastic therapy (41). The exosome is a sort of a small vesicle. Both malignant and normal cells can secrete exosome into their microenvironment (42). The proteins and genetic materials can be transmitted by exosomes so that the phenotype of immune cells, endothelial cells, and recipient stromal cells can be transformed in both the remote and the local areas (43, 44). Since miR-34a was frequently disordered in many kinds of cancer tissues, it was regarded as a potential tumor suppressor miRNA (45, 46). Additionally, numerous targets of expressions which were observed in carcinogenesis and tumor progression were reported to be regulated by miR-34a, such as CD44, BCL2, NOTCH1, CDK4/6, MET, MYC, and many other molecules (47, 48). In our study, the expression level of miR-34a was discovered to be negatively correlated with the MELK expression level in ULMS cells. Confirmatory similar results were obtained in the exosomes of ULMS cells with MELK overexpression. Furthermore, the result of the luciferase assay indicated that IL6R is the target gene of miRNA-34a. In addition, in p53 mutated colorectal cancer cells, a conservative STAT3 binding site directly repressed miR-34a, which was suggested as a p53-independent expression of miR-34a in IL-6-induced epithelial-mesenchymal transition and invasion (49). However, the p53-dependent mechanism of miR-34a regulation was considerable for the restriction of tumor progression by restraining the IL-6R/STAT3/miR-34a feedback loop (49). In the current study, MELK overexpression was shown to induce increased p-JAK2 and p-STAT3 and then to decrease miRNA-34a expression, as seen in decreased miRNA-34a in the exosomes of ULMS cells with MELK overexpression. Similarly, p-JAK2 and p-STAT3 in macrophages were also increased when the macrophages were treated in the conditional medium of ULMS cells with MELK overexpression. Our results indicate that MELK reduces miRNA-34a expression so that miRNA-34a is also decreased in the exosomes absorbed by the macrophages.

Decreased miRNA-34a can also induce the activation of JAK2/STAT3 pathway, which promotes M2 macrophage polarization. Our data show that IL-6 expression was increased in M2 macrophages. IL-6 is a multifaceted cytokine. IL-6 not only regulates reaction to infection or injury but also relates to immune disorders and cancers (50–52). The function of IL-6 has been explored in facilitating chemoresistance in various cancers in previous studies (50, 53, 54). We found that IL-6 could also promote chemoresistance to doxorubicin in ULMS cells. Additionally, M2 macrophages are important inflammatory cells in a tumor microenvironment. A major pathogenic activity of M2 macrophages is the immunosuppressive response. Tumor cells prevented from immunosurveillance is critical in tumor metastasis, survival, and growth. M2 macrophages could secrete immunosuppressive factors in a tumor microenvironment so that they could inhibit the immune response of T cells and manage the weak antigen-presenting capability. Prostaglandin E2 (PGE2), IL-10, TGF-β, and other immunosuppressive factors are also released by M2 macrophages. These factors recruit TReg cells, a cluster of immunodepressive T cells that can inhibit antitumor immune responses (55, 56). Moreover, TGF-β, as a momentous immunosuppressive factor, participates not only in innate immune response but also in adaptive immune response (57). The expression of several cytolytic genes in cytotoxic T lymphocytes (CTLs) could be suppressed by TGF-β, consisting of FAS ligand, IFN-γ, and genes encoding granzyme A and granzyme B, so that TGF-β could directly depress the function of CTL (58). NF-κB activation was suppressed by TGF-β, which also favors M2 macrophage polarization. Additionally, M2 macrophages engaged to the tumor by TGF-β may also secrete amounts of TGF-β in a vicious cycle. Furthermore, IL-10, a multifaceted cytokine secreted by M2 macrophages, could also induce immune depression. TAMs have been confirmed to produce a mass of IL-10 which can inhibit not only TH1 cell activity with a resultant suppression of CTL generation and activity but also NK and lymphokine-activated killer cell cytotoxicity (59). Moreover, antigen presentation and dendritic cell (DC) activity were also mediated by IL-10, which was also identified as another major approach by which IL-10 participated in preventing anti-tumor responses in the TME. The study also indicated that DC recruitment at the position of tumor cell inoculation could be depressed by IL-10, thus preventing DCs' exposure to tumor antigens (60). All in all, TAMs are appreciated to release mediators which are pivotal in immunosuppression. These mediators, combined with other immunosuppressive inflammatory cytokines released by tumor cells, engender immune damnification in the TME, which is the eventual result. This phenomenon could easily contribute to tumor cell survival and, ultimately, chemoresistance to cytotoxic drug effects.

This study represents a comprehensive analysis of MELK in ULMS chemoresistance. Our findings convey insights into the molecular mechanism wherein MELK induces chemoresistance via the JAK2/STAT3 pathway in ULMS cells. Our work also reveals that MELK can promote M2 macrophage polarization though the same pathway, which can then contribute to the chemoresistant property of ULMS cells (Figure 5E). Finally, considering ongoing or completed clinical trials on the effectiveness of the MELK inhibitor OTSSP167 in multiple tumors (NCT02926690, NCT01910545, and NCT02795520), the authors of this study suggest that a MELK inhibitor may contribute to doxorubicin's therapeutic effect when treatment is comprised of doxorubicin combined with OTSSP167. This concept warrants further investigation.

## Data Availability Statement

The mRNA/miRNA sequencing data analyzed in this manuscript has been uploaded to Sequence Read Archive (SRA). The accession number is SRR11249953, SRR11249952, SRR11249951, SRR11249950, SRR11249949, SRR11249948, SRR11249947, SRR11249946.

## Ethics Statement

The studies involving human participants were reviewed and approved by Ethics Committee of Shandong University. The patients/participants provided their written informed consent to participate in this study. The animal study was reviewed and approved by Shandong University Animal Care and Use Committee. Written informed consent was obtained from the individual(s) for the publication of any potentially identifiable images or data included in this article.

## Author Contributions

This study was conceived, designed, and interpreted by QZ and BK. ZZ, CS, and XJ undertook the data acquisition, analysis, and interpretation. XY and RD were responsible for the comprehensive technical support. ZZ, QZ, and SD were major contributors in writing the manuscript. XY and WC provided the ULMS samples. HW, CL, and CZ contributed to the inspection of data and final manuscript. BG contributed to the final manuscript. All authors read and approved the final manuscript.

## Conflict of Interest

The authors declare that the research was conducted in the absence of any commercial or financial relationships that could be construed as a potential conflict of interest.

## Supplementary Material

Supplementary Table 1 Clinicopathological characteristics in uterine LMS patients.
